# Supplementary material for: Hybrid Models and Biological Model Reduction with PyDSTool
Source: PLoS Comput Biol. 2012 Aug 9;8(8):e1002628. doi: 10.1371/journal.pcbi.1002628 (PMC3415397; doi:10.1371/journal.pcbi.1002628)
Supplement: Text S4 — Complete source code for the PyDSTool package (version 0.88.120504). Includes API documentation and help files linking to web pages. This file is identical to the current public release on Sourceforge.net. (ZIP) [file pcbi.1002628.s004.zip › PyDSTool/html/PyDSTool.Variable'-pysrc.html]

xml version="1.0" encoding="ascii"?


PyDSTool.Variable'


| Home | Trees | Indices | Help | PyDSTool |
| --- | --- | --- | --- | --- |

Package PyDSTool ::
Module Variable'

[hide private]

[frames] | no frames]

# Source Code for Module PyDSTool.Variable'

```
   1  """Variable is a one-dimensional discrete and continuous real variable class.
 
   2  
 
   3     Robert Clewley, July 2005
 
   4  """ 
   5  
 
   6  # ----------------------------------------------------------------------------
 
   7  
 
   8  # PyDSTool imports
 
   9  from utils import * 
  10  from common import * 
  11  from errors import * 
  12  from Points import * 
  13  from Interval import * 
  14  from FuncSpec import ImpFuncSpec 
  15  
 
  16  from numpy import Inf, NaN, isfinite, sometrue, alltrue, any, all, \
 
  17       array, float64, int32, ndarray, asarray 
  18  
 
  19  import copy 
  20  import types, math, random 
  21  
 
  22  __all__ = ['Variable', 'HybridVariable',
 
  23             'OutputFn', 'isinputcts', 'isinputdiscrete',
 
  24             'isoutputcts', 'isoutputdiscrete',
 
  25             'iscontinuous', 'isdiscrete',
 
  26             'numeric_to_vars', 'pointset_to_vars'] 
  27  
 
  28  # ------------------------------------------------------------------
 
  29  
 
  30  
 


31 -class VarDiagnostics(Diagnostics):


32 -    def getWarnings(self):


33          if self.warnings != []: 
  34              output = "Warnings:" 
  35              for (i,d) in self.warnings: 
  36                  if d is None: 
  37                      output += "Independent variable value %s was out of "% i + \
 
  38                            "bounds" 
  39                  else: 
  40                      output += "Dependent variable value %s was out of " % s + \
 
  41                            "bounds at independent variable value %s" % i 
  42          else: 
  43              output = '' 
  44          return output

  45  
 
  46  
 


47 -def pointset_to_vars(pts, discrete=True):


48      """Utility to convert Pointset to a dictionary of Variables.
 
  49      If discrete option set to False (default is True) then the
 
  50      Variables will be linearly interpolated within their domain.
 
  51  
 
  52      Any labels in the pointset will be preserved in the Variables
 
  53      in case of their re-extraction using the getDataPoints method.
 
  54      """ 
  55      coordnames = pts.coordnames 
  56      vals = pts.coordarray 
  57      all_types_float = pts.coordtype == float 
  58      if isparameterized(pts): 
  59          indepvar = pts.indepvararray 
  60          indepvarname = pts.indepvarname 
  61          if discrete: 
  62              indepvartype = int 
  63          else: 
  64              indepvartype = float 
  65          indepdomain = Interval(pts.indepvarname, indepvartype,
 
  66                                 extent(pts.indepvararray),
 
  67                                 abseps=pts._abseps) 
  68      else: 
  69          indepvar = None 
  70          indepvarname = None 
  71          indepdomain = None 
  72      return numeric_to_vars(vals, coordnames, indepvar, indepvarname,
 
  73                             indepdomain, all_types_float, discrete,
 
  74                             pts._abseps, pts.labels)

  75  
 
  76  
 


77 -def numeric_to_vars(vals, coordnames, indepvar=None, indepvarname='t',
 
  78                      indepdomain=None, all_types_float=True, discrete=True,
 
  79                      abseps=None, labels=None):


80      """Utility to convert numeric types to a dictionary of Variables.
 
  81      If discrete option set to True (default is False) then the
 
  82      Variables will be linearly interpolated within their domain.
 
  83      """ 
  84      if isinstance(coordnames, str): 
  85          coordnames = [coordnames] 
  86      if isinstance(vals, _num_types): 
  87          vals = [[vals]] 
  88      vars = {} 
  89      if indepvar is None: 
  90          for i, c in enumerate(coordnames): 
  91              if all_types_float: 
  92                  vartype = float 
  93              else: 
  94                  vartype = array(vals[i]).dtype.type 
  95              if discrete: 
  96                  vars[c] = Variable(outputdata=Pointset({'coordnames': c,
 
  97                                                      'coordarray': vals[i],
 
  98                                                      'coordtype': vartype}),
 
  99                                                      name=c, abseps=abseps,
 
 100                                                      labels=labels) 
 101              else: 
 102                  raise AssertionError("Cannot use continuously defined "
 
 103                                       "option without an independent variable") 
 104          return vars 
 105      else: 
 106          if isinstance(indepvar, _num_types): 
 107              indepvartype = type(indepvar) 
 108              indepvar = [indepvar] 
 109          else: 
 110              indepvartype = asarray(indepvar).dtype.type 
 111          if indepdomain is None: 
 112              indepdomain = indepvarname 
 113          else: 
 114              if isinstance(indepdomain, Interval): 
 115                  assert indepvarname == indepdomain.name, "Indep varname mismatch" 
 116              else: 
 117                  if discrete: 
 118                      var_type = int 
 119                  else: 
 120                      var_type = float 
 121                  indepdomain = Interval(indepvarname, var_type, indepdomain) 
 122          for i, c in enumerate(coordnames): 
 123              if all_types_float: 
 124                  vartype = float 
 125              else: 
 126                  vartype = array(vals[i]).dtype.type 
 127              if discrete: 
 128                  vars[c] = Variable(outputdata=Pointset({'coordnames': c,
 
 129                                                  'coordarray': vals[i],
 
 130                                                  'coordtype': vartype,
 
 131                                                  'indepvarname': indepvarname,
 
 132                                                  'indepvararray': indepvar,
 
 133                                                  'indepvartype': indepvartype}),
 
 134                                      indepdomain=indepdomain, name=c,
 
 135                                      abseps=abseps, labels=labels) 
 136              else: 
 137                  dom_int = Interval(c, vartype, extent(vals[i]),
 
 138                                     abseps=abseps) 
 139                  vars[c] = Variable(outputdata=interp1d(indepvar, vals[i]),
 
 140                                     indepdomain=indepdomain,
 
 141                                     depdomain=dom_int, name=c,
 
 142                                     abseps=abseps, labels=labels) 
 143      return vars

 144  
 
 145  
 


146 -class Variable(object):


147      """One-dimensional discrete and continuous real variable class.
 
 148      """ 
 149  
 


150 -    def __init__(self, outputdata=None, indepdomain=None, depdomain=None,
 
 151                   name='noname', abseps=None, labels=None):


152          # funcreg stores function data for dynamically created methods
 
 153          # to allow a Variable to be copied using pickling
 
 154          self._funcreg = {} 
 155          if isinstance(name, str): 
 156              # !!! name is probably redundant
 
 157              self.name = name 
 158          else: 
 159              raise TypeError("name argument must be a string") 
 160          # defaults for empty 'placeholder' Variables used by ODEsystem
 
 161          if outputdata is None or isinstance(outputdata, (Pointset, interp1d)): 
 162              if indepdomain is None: 
 163                  indepdomain = 't' 
 164              if depdomain is None: 
 165                  depdomain = 'x' 
 166          # set some initial values so that can test if what changed
 
 167          # after calling setOutput()
 
 168          self._vectorizable = True 
 169          self.defined = False 
 170          self.indepdomain = None 
 171          self.indepvartype = None 
 172          self.indepvarname = None 
 173          self.depdomain = None 
 174          self.coordtype = None 
 175          self.coordname = None 
 176          self._refvars = None   # for use with ExplicitFnGen 
 177          # Ranges covered by the current trajectory held (if known)
 
 178          self.trajirange = None 
 179          self.trajdrange = None 
 180          # independent variable domain
 
 181          self.setIndepdomain(indepdomain, abseps) 
 182          # used internally, especially for "input" variables
 
 183          self._internal_t_offset = 0 
 184          # output function
 
 185          self.setOutput(outputdata, abseps) 
 186          # dependent variable domain
 
 187          self.setDepdomain(depdomain, abseps) 
 188          assert self.coordname != self.indepvarname, ("Independent variable "
 
 189                                  "name and coordinate name must be different") 
 190          self.diagnostics = VarDiagnostics() 
 191          # labels is for internal use in case Variable data is from a Pointset
 
 192          # that uses labels. This preserves them for getDataPoints method to
 
 193          # restore them.
 
 194          self.labels = labels

 195  
 
 196  
 


197 -    def is_continuous_valued(self):


198          return isoutputcts(self)

 199  
 


200 -    def is_discrete_valued(self):


201          return not isoutputcts(self)

 202  
 
 203  
 
 204      # Auxiliary functions for user-defined code to call
 


205 -    def _auxfn_globalindepvar(self, parsinps, t):


206          return self.globalt0 + t

 207  
 
 208  
 


209 -    def _auxfn_initcond(self, parsinps, varname):


210          return self.initialconditions[varname]

 211  
 
 212  
 


213 -    def _auxfn_heav(self, parsinps, x):


214          if x>0: 
 215              return 1 
 216          else: 
 217              return 0

 218  
 
 219  
 


220 -    def _auxfn_if(self, parsinps, c, e1, e2):


221          if c: 
 222              return e1 
 223          else: 
 224              return e2

 225  
 
 226  
 


227 -    def _auxfn_getindex(self, parsinps, varname):


228          return self._var_namemap[varname]

 229  
 
 230  
 


231 -    def addMethods(self, funcspec):


232          """Add dynamically-created methods to Veriable object""" 
 233  
 
 234          # Add the auxiliary function specs to this Variable's namespace
 
 235          for auxfnname in funcspec.auxfns: 
 236              fninfo = funcspec.auxfns[auxfnname] 
 237              if not hasattr(Variable, fninfo[1]): 
 238                  # user-defined auxiliary functions
 
 239                  # (built-ins are provided explicitly)
 
 240                  try: 
 241                      exec fninfo[0] in globals() 
 242                  except: 
 243                      print 'Error in supplied auxiliary function code' 
 244                      raise 
 245                  self._funcreg[fninfo[1]] = ('Variable', fninfo[0]) 
 246                  setattr(Variable, fninfo[1], eval(fninfo[1])) 
 247          # Add the spec function to this Variable's namespace
 
 248          fninfo_spec = funcspec.spec 
 249          if not hasattr(Variable, fninfo_spec[1]): 
 250              try: 
 251                  exec fninfo_spec[0] in globals() 
 252              except: 
 253                  print 'Error in supplied functional specification code' 
 254                  raise 
 255              self._funcreg[fninfo_spec[1]] = ('Variable', fninfo_spec[0]) 
 256              setattr(Variable, fninfo_spec[1], eval(fninfo_spec[1])) 
 257          # Add the auxiliary spec function (if present) to this Var's namespace
 
 258          if funcspec.auxspec: 
 259              fninfo_auxspec = funcspec.auxspec 
 260              if not hasattr(Variable, fninfo_auxspec[1]): 
 261                  try: 
 262                      exec fninfo_auxspec[0] in globals() 
 263                  except: 
 264                      print 'Error in supplied auxiliary variable code' 
 265                      raise 
 266                  self._funcreg[fninfo_auxspec[1]] = ('Variable', fninfo_auxspec[0]) 
 267                  setattr(Variable, fninfo_auxspec[1], eval(fninfo_auxspec[1])) 
 268          # For implicit functions
 
 269          if isinstance(funcspec, ImpFuncSpec): 
 270              impfn_name = funcspec.algparams['impfn_name'] 
 271              if funcspec.algparams['jac']: 
 272                  jac_str = "fprime=funcspec.algparams['jac']," 
 273              else: 
 274                  jac_str = "" 
 275              # Wrap spec fn like this as it has been set up as a
 
 276              # method, but want to call as regular function
 
 277              # *** ALSO *** spec fn has signature (ds, t, x, p)
 
 278              # but implicit function solvers expect
 
 279              # (x, t, p), so have to switch 1st and 2nd args here
 
 280              # after 'ds' filled with None
 
 281              if len(funcspec.vars) == 1: 
 282                  # dimension == 1, so have to make list output from spec
 
 283                  # into a scalar
 
 284                  # Also, scalar a1 needs to be put into list form for
 
 285                  # acceptance as x in spec fn
 
 286                  specfn_str = "lambda a1, a2, a3: " \
 
 287                    + fninfo_spec[1] \
 
 288                    + "(None, a2, [a1], a3)[0]" 
 289              else: 
 290                  # for dimension > 1 a1 will already be an array / list
 
 291                  specfn_str = "lambda a1, a2, a3: " \
 
 292                    + fninfo_spec[1] \
 
 293                    + "(None, a2, a1, a3)" 
 294              this_scope = globals()   # WE CHANGE GLOBALS() 
 295              this_scope.update({'funcspec': locals()['funcspec'],
 
 296                                 'fninfo_spec': locals()['fninfo_spec']}) 
 297              impfn_str = impfn_name + \
 
 298                  " = makeImplicitFunc(" + specfn_str + "," \
 
 299                  + jac_str + """x0=funcspec.algparams['x0'],
 
 300                                 extrafargs=(funcspec.algparams['pars'],),
 
 301                                 xtolval=funcspec.algparams['atol'],
 
 302                                 maxnumiter=funcspec.algparams['maxnumiter'],
 
 303                                 solmethod=funcspec.algparams['solvemethod'],
 
 304                                 standalone=False)""" 
 305              try: 
 306                  exec impfn_str in this_scope 
 307              except: 
 308                  print 'Error in supplied implicit function code' 
 309                  raise 
 310              # record special reference to the implicit fn,
 
 311              # as its a method of Variable (for delete method).
 
 312              self._funcreg['_impfn'] = (impfn_name, impfn_str) 
 313              # In previous versions setattr was to self, not the Variable class
 
 314              setattr(Variable, impfn_name, eval(impfn_name)) 
 315              # clean up globals() afterwards
 
 316              del this_scope['funcspec'] 
 317              del this_scope['fninfo_spec']

 318  
 


319 -    def getDataPoints(self):


320          """Reveal underlying mesh and values at mesh points, provided
 
 321          Variable is based on a mesh (otherwise None is returned).
 
 322          The returned Pointset will be time-shifted according to the
 
 323          Variable's current _internal_t_offset attribute.
 
 324  
 
 325          Any pointset labels present when the variable was created will
 
 326          be restored.
 
 327          """ 
 328          if isinstance(self.output, VarCaller): 
 329              return Pointset(indepvarname=self.indepvarname,
 
 330                              indepvararray=self.output.pts.indepvararray + self._internal_t_offset,
 
 331                              coordnames=[self.coordname],
 
 332                              coordarray=self.output.pts.coordarray[0],
 
 333                              labels=self.labels) 
 334          elif hasattr(self.output, 'datapoints'): 
 335              datapoints = self.output.datapoints 
 336              return Pointset(indepvarname=self.indepvarname,
 
 337                              indepvararray=datapoints[0] + self._internal_t_offset,
 
 338                              coordnames=[self.coordname],
 
 339                              coordarray=datapoints[1],
 
 340                              labels=self.labels) 
 341          else: 
 342              return None

 343  
 


344 -    def underlyingMesh(self):


345          """Reveal underlying mesh as arrays, rather than Pointset
 
 346          as returned by getDataPoints method. If no underlying mesh is
 
 347          present, None is returned.""" 
 348          try: 
 349              # works if .output is an interpclass instance
 
 350              mesh = self.output.datapoints 
 351          except AttributeError: 
 352              try: 
 353                  # works if .output is a VarCaller instance (with underlying Pointset)
 
 354                  pts = self.output.pts 
 355                  mesh = array([pts.indepvararray, pts.coordarray[0]]) 
 356              except AttributeError: 
 357                  mesh = None 
 358          return mesh

 359  
 


360 -    def truncate_to_idx(self, idx):


361          mesh = self.underlyingMesh() 
 362          if mesh is None: 
 363              raise RuntimeError("Cannot truncate a Variable without an underlying mesh by index") 
 364          try: 
 365              new_t_end = mesh[0][idx] 
 366          except IndexError: 
 367              raise ValueError("Truncation index %d out of range"%idx) 
 368          except TypeError: 
 369              raise TypeError("Index must be an integer") 
 370          if isinstance(self.indepdomain, Interval): 
 371              self.indepdomain.set([self.indepdomain[0], new_t_end]) 
 372          else: 
 373              # ndarray type
 
 374              self.indepdomain = self.indepdomain[0:idx] 
 375          # adjust depdomain for array type of dep domain
 
 376          # (nothing to change for Interval type)
 
 377          if isinstance(self.depdomain, ndarray): 
 378              self.depdomain = self.depdomain[0:idx] 
 379          # adjust trajirange and trajdrange
 
 380          self._setRanges(self.depdomain._abseps)

 381  
 


382 -    def _setRanges(self, abseps=None):


383          # set trajirange and trajdrange for the two types of Variable output method
 
 384          # that these are associated with (see method setOutput)
 
 385          try: 
 386              output = self.output 
 387          except AttributeError: 
 388              # output not set or not a compatible type for trajirange and trajdrange
 
 389              return 
 390          if isinstance(output, VarCaller): 
 391              self.trajirange = Interval('traj_indep_bd',
 
 392                                         self.indepvartype,
 
 393                                         extent(output.pts.indepvararray),
 
 394                                     abseps=abseps) 
 395              self.trajdrange = Interval('traj_dep_bd',
 
 396                                         self.coordtype,
 
 397                                         extent(output.pts.coordarray[0]),
 
 398                                     abseps=abseps) 
 399          elif type(output) in [types.InstanceType, types.TypeType] \
 
 400                                 or isinstance(output, (OutputFn, interpclass)): 
 401              if hasattr(output, 'types'): 
 402                  deptype = output.types[0] 
 403                  indeptype = output.types[1] 
 404              else: 
 405                  # default
 
 406                  deptype = indeptype = float 
 407              if isinstance(output.datapoints[0], Interval): 
 408                  assert compareNumTypes(output.types[0], \
 
 409                         output.datapoints[0].type), \
 
 410                         "Inconsistent type with Interval bounds" 
 411                  self.trajirange = output.datapoints[0] 
 412              else: 
 413                  self.trajirange = Interval('traj_indep_bd', indeptype,
 
 414                                            extent(output.datapoints[0]),
 
 415                                        abseps=abseps) 
 416              if isinstance(output.datapoints[1], Interval): 
 417                  assert compareNumTypes(output.types[1], \
 
 418                         output.datapoints[1].type), \
 
 419                         "Inconsistent type with Interval bounds" 
 420                  self.trajdrange = output.datapoints[1] 
 421              else: 
 422                  self.trajdrange = Interval('traj_dep_bd', deptype,
 
 423                                            extent(output.datapoints[1]),
 
 424                                        abseps=abseps)

 425  
 


426 -    def setOutput(self, outputdata, funcspec=None, globalt0=0,
 
 427                    var_namemap=None, ics=None, refvars=None, abseps=None):


428          """Dynamically create 'output' method of Variable""" 
 429  
 
 430          self.globalt0 = globalt0 
 431          if type(outputdata) in [types.FunctionType,
 
 432                                  types.BuiltinFunctionType,
 
 433                                  types.MethodType]: 
 434              # Variable generated from function, given in closed form
 
 435              self.output = outputdata 
 436              assert ics is None, "Invalid option for this type of output" 
 437              if outputdata != noneFn: 
 438                  self.defined = True 
 439          elif isinstance(outputdata, tuple): 
 440              # For ExplicitFnGen or ImplicitFnGen types, whose functional forms
 
 441              # may need to access these at call time.
 
 442              assert len(outputdata) == 2, "Incorrect size of outputdata tuple" 
 443              if funcspec is not None: 
 444                  self.addMethods(funcspec) 
 445                  self._var_namemap = var_namemap 
 446                  self._funcreg['funcspec'] = (None, funcspec) 
 447              else: 
 448                  raise ValueError('funcspec missing in setOutput') 
 449              # Add the specific mapping functions for Ex/ImplicitFnGen objects
 
 450              try: 
 451                  exec outputdata[1] in globals() 
 452              except: 
 453                  print 'Internal Error in _mapspecfn code' 
 454                  raise 
 455              has_op = hasattr(self, 'output') 
 456              # have to define this function in here because use of lambda
 
 457              # won't allow me to pickle the Variable object
 
 458              if not has_op or (has_op and self.output is noneFn): 
 459                  def wrap_output(arg): 
 460                      return eval(outputdata[0])(self, arg)

 461                  setattr(self, 'output', wrap_output) 
 462              self._funcreg['outputdata'] = (None, outputdata) 
 463              t0 = self.indepdomain[0] 
 464              if ics is None and not isinstance(funcspec, ImpFuncSpec): 
 465                  try: 
 466                      self.initialconditions = {self.coordname: self.output(t0)} 
 467                  except ValueError: 
 468                      self.initialconditions = {self.coordname: NaN} 
 469                  except TypeError: 
 470                      print "Debugging info: self.output = ", self.output 
 471                      raise 
 472              else: 
 473                  self.initialconditions = ics 
 474              self._vectorizable = False 
 475              self._refvars = refvars 
 476              self.defined = True 
 477          elif type(outputdata) in [types.InstanceType, types.TypeType] \
 
 478                               or isinstance(outputdata, (OutputFn, interpclass)): 
 479              # Variable generated by callable object that generates values over
 
 480              # mesh points that it holds, e.g. by interpolation
 
 481              # (InstanceType and TypeType are for backwards compatibility, e.g.
 
 482              # for old SciPy interpolate code that uses Classic Classes)
 
 483              assert ics is None, "Invalid option for this type of output" 
 484              assert '__call__' in dir(outputdata), "Must provide callable object" 
 485              self.output = outputdata 
 486              if hasattr(outputdata, 'datapoints'): 
 487                  self._setRanges(abseps) 
 488              self.defined = True 
 489          elif isinstance(outputdata, Pointset): 
 490              # Variable generated from a pointset (without interpolation)
 
 491              assert ics is None, "Invalid option for this type of output" 
 492              assert isparameterized(outputdata), ("Must only pass parameterized"
 
 493                                                   " pointsets") 
 494              if outputdata.dimension == 1: 
 495                  self.coordname = copy.copy(outputdata.coordnames[0]) 
 496                  self.indepvarname = outputdata.indepvarname 
 497                  self.output = VarCaller(outputdata) 
 498                  self.coordtype = outputdata.coordtype 
 499                  self.indepvartype = outputdata.indepvartype 
 500                  if self.indepdomain is not None: 
 501                      for v in outputdata[self.indepvarname]: 
 502                          if not v in self.indepdomain: 
 503                              raise ValueError("New Pointset data violates "
 
 504                                 "independent variable domain already specified") 
 505                  if self.depdomain is not None: 
 506                      for v in outputdata[self.coordname]: 
 507                          if not v in self.depdomain: 
 508                              raise ValueError("New Pointset data violates "
 
 509                                 "dependent variable domain already specified") 
 510                  self._setRanges(abseps) 
 511                  self.defined = True 
 512              else: 
 513                  raise ValueError("Pointset data must be 1D to create a "
 
 514                                   "Variable") 
 515          elif outputdata is None: 
 516              # placeholder for an unknown output type
 
 517              assert ics is None, "Invalid option when outputdata argument is None" 
 518              self.output = noneFn 
 519              self.defined = False 
 520          else: 
 521              raise TypeError("Invalid type for data argument: " \
 
 522                                +str(type(outputdata)))

 523  
 
 524  
 


525 -    def setIndepdomain(self, indepdomain, abseps=None):


526          if isinstance(indepdomain, str): 
 527              self.indepvarname = indepdomain 
 528              if self.indepdomain is not None: 
 529                  # If indepdomain already set and indepvarname is none then
 
 530                  # name won't get put in place unless we force it here
 
 531                  self.indepvarname = indepdomain 
 532                  self.indepdomain.name = indepdomain 
 533              else: 
 534                  self.indepdomain = Interval(self.indepvarname, float,
 
 535                                             [-Inf, Inf], abseps=abseps) 
 536              self.indepvartype = float 
 537          else: 
 538              if isinstance(indepdomain, Interval): 
 539                  if self.trajirange: 
 540                      if indepdomain.contains(self.trajirange) is notcontained: 
 541                          raise ValueError("Cannot set independent variable"
 
 542                                           " domain inside current trajectory's"
 
 543                                           " range") 
 544                  self.indepdomain = indepdomain 
 545                  self.indepvarname = indepdomain.name 
 546                  self.indepvartype = _num_name2type[indepdomain.typestr] 
 547              elif isinstance(indepdomain, dict): 
 548                  # enumerated discrete domains
 
 549                  assert len(indepdomain) == 1, "Independent variable " \
 
 550                                           "dictionary must have only 1 entry" 
 551                  d = indepdomain.values()[0] 
 552                  assert all(isfinite(d)), "Independent variable values must be" \
 
 553                                           " finite" 
 554                  if self.trajirange: 
 555                      assert self.trajirange[0] in d 
 556                      assert self.trajirange[1] in d 
 557                  self.indepvarname = indepdomain.keys()[0] 
 558                  if isinstance(d, (list, tuple)): 
 559                      if self.coordtype is not None: 
 560                          self.indepdomain = array(d, self.coordtype) 
 561                      else: 
 562                          self.indepdomain = array(d) 
 563                  elif isinstance(d, ndarray): 
 564                      da = array(d) 
 565                      if self.indepvartype is not None and \
 
 566                         self.indepvartype != da.dtype.type: 
 567                          raise TypeError("Mismatch between type of indepdomain "
 
 568                                            "argument and Pointset data") 
 569                      else: 
 570                          self.indepdomain = da 
 571                  else: 
 572                      raise TypeError("Invalid type for independent "
 
 573                                        "variable domain") 
 574                  # assert this after self.indepdomain has been made an array
 
 575                  # because isincreasing is most efficient on already-created
 
 576                  # arrays
 
 577                  assert isincreasing(self.indepdomain), \
 
 578                         "Independent variable values must be increasing" 
 579                  self.indepvartype = self.indepdomain.dtype.type 
 580              else: 
 581                  print "Independent variable argument domain was:", indepdomain 
 582                  raise TypeError("Invalid type for independent variable "
 
 583                                    "domain")

 584  
 
 585  
 


586 -    def setDepdomain(self, depdomain, abseps=None):


587          if isinstance(depdomain, str): 
 588              self.coordname = depdomain 
 589              if self.depdomain is None: 
 590                  if self.coordtype is None: 
 591                      self.depdomain = Interval(self.coordname, float,
 
 592                                                   [-Inf, Inf], abseps=abseps) 
 593                      self.coordtype = float 
 594                  else: 
 595                      self.depdomain = Interval(self.coordname,
 
 596                                                   self.coordtype,
 
 597                                                   _num_maxmin[self.coordtype],
 
 598                                               abseps=abseps) 
 599              else: 
 600                  # If interp functions supplied then don't have a name for
 
 601                  # Interval yet, so update it.
 
 602                  if isinstance(self.output, interpclass) and \
 
 603                     isinstance(self.depdomain, Interval): 
 604                      self.depdomain.name = depdomain 
 605                  else: 
 606                      assert isinstance(self.output, Pointset) 
 607                      self.diagnostics.warnings.append((self.depdomain.name,
 
 608                                            "Dependent variable already named. "
 
 609                                            "Ignoring user-supplied name.")) 
 610          else: 
 611              if isinstance(depdomain, Interval): 
 612                  if self.trajdrange: 
 613                      if depdomain.contains(self.trajdrange) is notcontained: 
 614                          raise ValueError("Cannot set dependent variable "
 
 615                                            "domain inside current trajectory's "
 
 616                                            "range") 
 617                  self.depdomain = depdomain 
 618                  self.coordname = depdomain.name 
 619                  if self.coordtype is None: 
 620                      self.coordtype = depdomain.type 
 621                  elif self.coordtype == depdomain.type: 
 622                      pass 
 623                  else: 
 624                      raise TypeError("Mismatch between type of depdomain "
 
 625                                        "argument and Pointset coord data") 
 626              elif isinstance(depdomain, dict): 
 627                  assert len(depdomain) == 1, \
 
 628                         "Depend variables dictionary must have only 1 entry" 
 629                  d = depdomain.values()[0] 
 630                  if self.trajdrange: 
 631                      assert self.trajdrange[0] in d 
 632                      assert self.trajdrange[1] in d 
 633                  ## Assume d is in increasing order
 
 634                  assert all(isfinite(d)), "Values must be finite" 
 635                  self.coordname = depdomain.keys()[0] 
 636                  if isinstance(d, (list, tuple)): 
 637                      if self.coordtype is not None: 
 638                          self.depdomain = array(d, self.coordtype) 
 639                      else: 
 640                          self.depdomain = array(d) 
 641                  elif isinstance(d, ndarray): 
 642                      da = array(d) 
 643                      if self.coordtype is not None and \
 
 644                         self.coordtype != da.dtype.type: 
 645                          raise TypeError("Mismatch between type of depdomain "
 
 646                                            "argument and Pointset coord data") 
 647                      else: 
 648                          self.depdomain = da 
 649                  else: 
 650                      raise TypeError("Invalid type for dependent variable "
 
 651                                        "domain") 
 652                  self.coordtype = self.depdomain.dtype.type 
 653              else: 
 654                  print "Dependent variable domain argument was:", depdomain 
 655                  raise TypeError("Invalid type for dependent variable domain") 
 656              if isinstance(self.output, Pointset): 
 657                  assert self.coordname == self.output.coordnames[0], \
 
 658                         "Mismatch between Pointset coord name and declared name" 
 659                  assert self.indepvarname == self.output.indepvarname, \
 
 660                         ("Mismatch between Pointset independent variable name "
 
 661                          "and declared name")

 662  
 
 663  
 


664 -    def __call__(self, indepvar, checklevel=0):


665          # Set actual time by subtracting internal offset. Especially for use by
 
 666          # "input" variables that are based on inherently time-shifted
 
 667          # arrays of values, with nothing to do with the globalt0 of hybrid
 
 668          # trajectories.
 
 669          indepvar = asarray(indepvar) - self._internal_t_offset 
 670          if checklevel == 0: 
 671              # level 0 -- no depvar bounds checking at all
 
 672              # (no need to check for indepvar as list case, which output
 
 673              # should know how to handle)
 
 674              try: 
 675                  if not self._vectorizable and isinstance(indepvar, _seq_types): 
 676                      return [self.output(ival) for ival in indepvar] 
 677                  else: 
 678                      return self.output(indepvar) 
 679              except (OverflowError, ValueError): 
 680                  self.diagnostics.errors.append((indepvar, self.name + ": Overflow error in output")) 
 681                  raise 
 682              except PyDSTool_BoundsError: 
 683                  self.diagnostics.errors.append((indepvar, self.name + ": Bounds error in output")) 
 684                  raise 
 685          elif checklevel in [1,2]: 
 686              if self.trajirange is None: 
 687                  idep = self.indepdomain 
 688              else: 
 689                  # use known bounds on indep variable imposed by self.output
 
 690                  idep = self.trajirange 
 691              indepvar_ok = True 
 692              # level 1 -- ignore uncertain cases (treat as contained)
 
 693              # level 2 -- warn on uncertain (treat as contained)
 
 694              if isinstance(indepvar, _seq_types): 
 695                  vectorizable = self._vectorizable 
 696                  for d in indepvar: 
 697                      # use 'in' so that this is compatible with
 
 698                      # interval, array and index indeps
 
 699                      try: 
 700                          contresult = d in idep 
 701                      except PyDSTool_UncertainValueError: 
 702                          contresult = True 
 703                          # adjust for rounding error so that interpolator
 
 704                          # does not barf on out-of-range values
 
 705                          if d < idep[0]: 
 706                              try: 
 707                                  # list
 
 708                                  dix = indepvar.index(d) 
 709                              except AttributeError: 
 710                                  # array
 
 711                                  dix = indepvar.tolist().index(d) 
 712                              indepvar[dix] = idep[0] 
 713                          elif d > idep[1]: 
 714                              try: 
 715                                  # list
 
 716                                  dix = indepvar.index(d) 
 717                              except AttributeError: 
 718                                  # array
 
 719                                  dix = indepvar.tolist().index(d) 
 720                              indepvar[dix] = idep[1] 
 721                          if checklevel == 2: 
 722                              self.diagnostics.warnings.append((d, None)) 
 723                      if not contresult: 
 724                          indepvar_ok = False 
 725                          break 
 726              else: 
 727                  vectorizable = True 
 728                  try: 
 729                      indepvar_ok = indepvar in idep 
 730                  except PyDSTool_UncertainValueError, errinfo: 
 731                      # adjust for rounding error so that interpolator
 
 732                      # does not barf on out-of-range values
 
 733                      if indepvar < idep[0]: 
 734                          indepvar = idep[0] 
 735                      elif indepvar > idep[1]: 
 736                          indepvar = idep[1] 
 737                      if checklevel == 2: 
 738                          self.diagnostics.warnings.append((indepvar, None)) 
 739              # continue to get dependent variable value, unless indep
 
 740              # value was not OK
 
 741              if not indepvar_ok: 
 742  ##                print "*** Debug info for variable: ", self.name
 
 743  ##                print "Interval rounding tolerance was", idep._abseps
 
 744                  if checklevel == 2: 
 745                      self.diagnostics.errors.append((indepvar,
 
 746                                  self.name + " : " + self.indepdomain._infostr(1))) 
 747                  if vectorizable: 
 748                      raise ValueError('Independent variable value(s) '
 
 749                                         'out of range in Variable call') 
 750                  else: 
 751                      raise ValueError('Independent variable value '+\
 
 752                                     str(indepvar) + ' out of '
 
 753                                     'range in Variable call') 
 754              try: 
 755                  if vectorizable: 
 756                      depvar = self.output(indepvar) 
 757                  else: 
 758                      depvar = [self.output(ival) for ival in indepvar] 
 759                  depvar_ok = True 
 760              except PyDSTool_BoundsError, errinfo: 
 761                  depvar_ok = False 
 762              # Now check that all computed values were in depdomain
 
 763              if depvar_ok: 
 764                  # no need to use self.trajdrange instead of
 
 765                  # self.depdomain because we trust that self.output
 
 766                  # generated the output within its own bounds!
 
 767                  if isinstance(depvar, (_seq_types, Pointset)): 
 768                      if isinstance(depvar, Pointset): 
 769                          dv = depvar.toarray() 
 770                      else: 
 771                          dv = depvar 
 772                      for d in dv: 
 773                          # use 'in' so that this is compatible with
 
 774                          # interval, array and index indeps
 
 775                          try: 
 776                              contresult = d in self.depdomain 
 777                          except PyDSTool_UncertainValueError, errinfo: 
 778                              contresult = True 
 779                              if checklevel == 2: 
 780                                  # find which indepvar was the cause of
 
 781                                  # the uncertain value
 
 782                                  try: 
 783                                      # list
 
 784                                      depix = dv.index(d) 
 785                                  except AttributeError: 
 786                                      # array
 
 787                                      depix = dv.tolist().index(d) 
 788                                  self.diagnostics.warnings.append((indepvar[depix], errinfo.value)) 
 789                          if not isfinite(d): 
 790                              # DEBUG
 
 791                              #print dv
 
 792                              #print self.output, "\n"
 
 793                              raise PyDSTool_BoundsError("Return value was not finite/defined (%s)"%str(d)) 
 794                          if not contresult: 
 795                              depvar_ok = False 
 796                              break 
 797                  elif depvar is None: 
 798                      # DEBUG
 
 799                      #print "*** Debug info for variable: ", self.name
 
 800                      #print "Independent variable domain: ", self.indepdomain._infostr(1)
 
 801                      #print "Dependent variable domain: ", self.depdomain._infostr(1)
 
 802                      raise ValueError("Cannot compute a return value for "
 
 803                                            "independent variable value "
 
 804                                            + str(indepvar)) 
 805                  else: 
 806                      if isinstance(depvar, Point): 
 807                          dv = depvar[0] 
 808                      else: 
 809                          dv = depvar 
 810                      try: 
 811                          depvar_ok = dv in self.depdomain 
 812                      except PyDSTool_UncertainValueError, errinfo: 
 813                          if checklevel == 2: 
 814                              self.diagnostics.warnings.append((indepvar, errinfo.varval)) 
 815                      if not isfinite(dv): 
 816                          # DEBUG
 
 817                          #print dv
 
 818                          #print self.output, "\n"
 
 819                          raise PyDSTool_BoundsError("Return value was not finite/defined (%s)"%str(dv)) 
 820              # return value if depvar in bounds
 
 821              if depvar_ok: 
 822                  return dv 
 823              else: 
 824                  # DEBUG
 
 825                  #print "Variable '%s' -"%self.name, "dependent var domain: ", \
 
 826                  #      self.depdomain._infostr(1)
 
 827                  #self.diagnostics.showWarnings()
 
 828                  if vectorizable: 
 829                      # DEBUG
 
 830                      #print self.output(indepvar), "\n"
 
 831                      raise PyDSTool_BoundsError('Computed value(s) %f outside'%dv + \
 
 832                                     ' validity range in Variable call') 
 833                  else: 
 834                      raise PyDSTool_BoundsError('Computed value %f outside'%dv + \
 
 835                                     ' validity range in Variable call') 
 836          else: 
 837              # level 3 -- exception will be raised for uncertain case
 
 838              indepvar_ok = False 
 839              try: 
 840                  # don't trap uncertain case exception from
 
 841                  # Interval.__contains__
 
 842                  if isinstance(indepvar, _seq_types): 
 843                      vectorizable = self._vectorizable 
 844                      indepvar_ok = all([i in self.indepdomain for i in \
 
 845                                             indepvar]) 
 846                  else: 
 847                      vectorizable = True 
 848                      indepvar_ok = indepvar in self.indepdomain 
 849              except TypeError, e: 
 850                  raise TypeError('Something messed up with the Variable '
 
 851                                    'initialization: '+str(e)) 
 852              else: 
 853                  if not indepvar_ok: 
 854                      raise ValueError('Independent variable '+str(indepvar)+\
 
 855                                         ' out of range in Variable call') 
 856              # Don't need 'if indepvar_ok' because exception would have
 
 857              # been raised.
 
 858              # For this checklevel, don't trap uncertain case exception from
 
 859              # Interval.__contains__
 
 860              try: 
 861                  if vectorizable: 
 862                      depvar = self.output(indepvar) 
 863                      depvar_ok = depvar in self.depdomain 
 864                  else: 
 865                      depvar = [self.output(ival) for ival in indepvar] 
 866                      depvar_ok = all([d in self.depdomain for d in \
 
 867                                           depvar]) 
 868              except PyDSTool_BoundsError, e: 
 869                  raise ValueError("Cannot compute a return value for "
 
 870                                        "this independent variable value: "
 
 871                                        + str(e)) 
 872              except PyDSTool_TypeError: 
 873                  if not self.defined: 
 874                      print "Variable '%s' not fully defined."%self.name 
 875                      return None 
 876                  else: 
 877                      raise 
 878              else: 
 879                  if depvar_ok: 
 880                      return depvar 
 881                  else: 
 882                      if vectorizable: 
 883                          raise PyDSTool_BoundsError('Computed value(s) '
 
 884                                      'outside validity range in Variable call') 
 885                      else: 
 886                          raise PyDSTool_BoundsError('Computed value '+str(depvar)+\
 
 887                                      'outside validity range in Variable call')

 888  
 
 889  
 


890 -    def __repr__(self):


891          return self._infostr(verbose=0)

 892  
 
 893  
 
 894      __str__ = __repr__ 
 895  
 
 896  
 


897 -    def _infostr(self, verbose=1):


898          if verbose == 0: 
 899              return "Variable "+self.coordname+"("+self.indepvarname+")" 
 900          else: 
 901              try: 
 902                  if isinputcts(self): 
 903                      ipstr = "continuous" 
 904                  else: 
 905                      ipstr = "discrete" 
 906              except ValueError: 
 907                  ipstr = "not defined" 
 908              outputStr = "Variable:\n  Independent variable '" \
 
 909                          + self.indepvarname + "' [" + ipstr + "]\n" 
 910              try: 
 911                  if isoutputcts(self): 
 912                      opstr = "continuous" 
 913                  else: 
 914                      opstr = "discrete" 
 915              except ValueError: 
 916                  opstr = "not defined" 
 917              outputStr += "    defined in domain  " + str(self.indepdomain) 
 918              if verbose == 2: 
 919                  if self.trajirange is None: 
 920                      outputStr += "\n    ranges not known for this trajectory" 
 921                  else: 
 922                      outputStr += "\n    trajectory ranges  "+str(self.trajirange) 
 923              outputStr += "\nDependent variable '" + self.coordname + \
 
 924                          "' [" + opstr + "]\n    defined in domain  " 
 925              if not isinstance(self.depdomain, Interval): 
 926                  outputStr += _num_type2name[self.coordtype]+": " 
 927              outputStr += str(self.depdomain) 
 928              if verbose == 2: 
 929                  if self.trajdrange is None: 
 930                      outputStr += "\n    ranges not known for this trajectory" 
 931                  else: 
 932                      outputStr += "\n    trajectory ranges  "+str(self.trajdrange) 
 933              return outputStr

 934  
 
 935  
 


936 -    def info(self, verboselevel=1):


937          print self._infostr(verboselevel)

 938  
 
 939  
 


940 -    def __copy__(self):


941          pickledself = pickle.dumps(self) 
 942          return pickle.loads(pickledself)

 943  
 
 944  
 


945 -    def __deepcopy__(self, memo=None, _nil=[]):


946          pickledself = pickle.dumps(self) 
 947          return pickle.loads(pickledself)

 948  
 
 949  
 


950 -    def __getstate__(self):


951          d = copy.copy(self.__dict__) 
 952          # remove reference to Cfunc types by converting to strings
 
 953          d['indepvartype'] = _num_type2name[self.indepvartype] 
 954          d['coordtype'] = _num_type2name[self.coordtype] 
 955          if 'funcspec' in self._funcreg: 
 956              # then self is Imp/ExplicitFnGen and 'output' could not
 
 957              # be put in _funcreg because it relies on wrap_output
 
 958              # function that's not in the global namespace (so pickle fails
 
 959              # to find it)
 
 960              del d['output'] 
 961          for fname, finfo in self._funcreg.iteritems(): 
 962              if finfo[0] == 'self': 
 963                  try: 
 964                      del d[fname] 
 965                  except KeyError: 
 966                      pass 
 967              # else it's a Variable class method which won't get pickled
 
 968              # anyway, and will be restored to any class not in possession
 
 969              # of it if this object is unpickled
 
 970          return d

 971  
 
 972  
 


973 -    def __setstate__(self, state):


974          self.__dict__.update(state) 
 975          #print self.name, "- setstate: self.depdomain = ", self.depdomain.get()
 
 976          # reinstate Cfunc types
 
 977          self.indepvartype = _num_name2type[self.indepvartype] 
 978          self.coordtype = _num_name2type[self.coordtype] 
 979          # reinstate dynamic methods / functions
 
 980          for fname, finfo in self._funcreg.iteritems(): 
 981              if finfo[0] == 'self' and not hasattr(eval(finfo[0]), fname): 
 982                  # avoids special entry for 'outputdata'
 
 983                  setattr(eval(finfo[0]), fname, finfo[1]) 
 984          if 'funcspec' in self._funcreg: 
 985              # Add the specific mapping functions for Ex/ImplicitFnGen objects
 
 986              funcspec = self._funcreg['funcspec'][1] 
 987              outputdata = self._funcreg['outputdata'][1] 
 988              if hasattr(self, '_var_namemap'): 
 989                  var_namemap = self._var_namemap 
 990              else: 
 991                  var_namemap = None 
 992              if hasattr(self, 'initialconditions'): 
 993                  ics = copy.copy(self.initialconditions) 
 994              else: 
 995                  ics = None 
 996              if hasattr(self, '_refvars'): 
 997                  if self._refvars is not None and self._refvars != []: 
 998                      refvars = [copy.copy(v) for v in self._refvars] 
 999                  else: 
1000                      refvars = None 
1001              else: 
1002                  refvars = None 
1003              # if refvars in dictionary then just leave them there!
 
1004              self.setOutput(outputdata, funcspec,
 
1005                self.globalt0, var_namemap, ics, refvars)

1006  
 
1007  
 


1008 -    def __del__(self):


1009          # delete object-specific class methods etc. before deleting
 
1010          # to avoid crowding namespace
 
1011  ##        if hasattr(self, 'output'):
 
1012  ##            del self.output
 
1013          for fname, finfo in self._funcreg.iteritems(): 
1014              # Treat special cases first
 
1015              if finfo[0] is None: 
1016                  # don't want to eval(None) below
 
1017                  continue 
1018              elif fname == '_impfn': 
1019                  exec_str = 'del Variable.' + finfo[0] 
1020                  try: 
1021                      exec exec_str 
1022                  except AttributeError: 
1023                      # Uncertain why the name appears multiple times for their
 
1024                      # to be multiple attempts to delete it (which of course
 
1025                      # fail after the first successful attempt)
 
1026                      pass 
1027              elif fname is 'funcspec': 
1028                  # doesn't refer to any dynamically-created methods
 
1029                  # so ignore
 
1030                  pass 
1031              elif fname is 'outputdata': 
1032                  # doesn't refer to any dynamically-created methods
 
1033                  # so ignore
 
1034                  pass 
1035              elif hasattr(eval(finfo[0]), fname): 
1036                  exec_str = 'del '+ finfo[0] + '.' + fname 
1037                  try: 
1038                      exec exec_str 
1039                  except RuntimeError: 
1040                      # sometimes get these when objects improperly delted
 
1041                      # and new objects with the same name created
 
1042                      pass 
1043          if hasattr(self, '_refvars'): 
1044                  if self._refvars is not None and self._refvars != []: 
1045                      for v in self._refvars: 
1046                          v.__del__()

1047  
 
1048  
 


1049 -class HybridVariable(Variable):


1050      """Mimics part of the API of a non-hybrid variable.
 
1051  
 
1052      This is a somewhat ugly hack as it's implemented by using a whole
 
1053      HybridTrajectory object to extract individual variable values,
 
1054      rather than having extracted a sequence of Variable objects from
 
1055      a HT and stitching them back together as a single entity.""" 


1056 -    def __init__(self, hybridtraj, coordname, indepdomain, abseps=None):


1057          # store reference to the hybrid trajectory
 
1058          self._ht = hybridtraj 
1059          self.name = 'Hybrid variable '+coordname 
1060          self.outputdata = None    # not used 
1061          self.defined = True 
1062          self.indepvarname = 't' 
1063          self.indepdomain = indepdomain 
1064          self.indepvartype = float 
1065          self.coordname = coordname 
1066          self.depdomain = Interval(self.coordname, float,
 
1067                                      [-Inf, Inf], abseps=abseps) 
1068          self.coordtype = float 
1069          self.trajirange = None 
1070          self.trajdrange = None 
1071          self.diagnostics = Diagnostics() 
1072          # important that this isn't a Pointset for Variable.py's
 
1073          # isinputcts, isoutputcts, etc.
 
1074          self.output = None

1075  
 


1076 -    def __call__(self, indepvar, checklevel=0):


1077          return self._ht(indepvar, self.coordname, checklevel=checklevel)

1078  
 


1079 -    def getDataPoints(self):


1080          """Returns a Pointset of independent and dependent variable values,
 
1081          provided variable is based on a mesh (otherwise None is returned).
 
1082          """ 
1083          return self._ht.sample([self.coordname])

1084  
 


1085 -    def underlyingMesh(self):


1086          """Reveal underlying mesh as arrays, rather than Pointset as returned
 
1087          by getDataPoints method.""" 
1088          vs = self._ht.sample([self.coordname]) 
1089          return array([vs.indepvararray, vs.coordarray[0]])

1090  
 


1091 -    def __repr__(self):


1092          return "Hybrid variable "+self.coordname

1093  
 
1094      __str__ = __repr__ 
1095  
 


1096 -    def info(self, verboselevel=1):


1097          return "Hybrid variable "+self.coordname

1098  
 
1099      # overrides from Variable class
 
1100  
 


1101 -    def __getstate__(self):


1102          return copy.copy(self.__dict__)

1103  
 


1104 -    def __setstate__(self, state):


1105          self.__dict__.update(state)

1106  
 


1107 -    def __del__(self):


1108          # must override Variable.__del__
 
1109          pass

1110  
 
1111  
 
1112  
 


1113 -class OutputFn(object):


1114      """One-dimensional function wrapper.""" 
1115  
 


1116 -    def __init__(self, fn, datapoints=None, numtypes=(float64,float64),
 
1117                   abseps=None):


1118          assert isinstance(fn, types.FunctionType) or \
 
1119                 isinstance(fn, types.BuiltinFunctionType), \
 
1120                 ("fn argument must be a regular Python function") 
1121          self.fn = fn 
1122          # datapoints can be exhaustive list of known values for fn or
 
1123          # a Interval range for continuous-valued functions
 
1124          if datapoints is None: 
1125              datapoints = (Interval('indepvardom', numtypes[0], [-Inf, Inf],
 
1126                                     abseps=abseps),
 
1127                            Interval('depvardom', numtypes[1], [-Inf, Inf],
 
1128                                 abseps=abseps)) 
1129          try: 
1130              self.datapoints = (datapoints[0], datapoints[1]) 
1131          except TypeError: 
1132              raise TypeError("datapoints argument must be a 2-tuple or list "
 
1133                                "of 2-tuples or lists") 
1134          try: 
1135              self.types = (numtypes[0], numtypes[1]) 
1136          except TypeError: 
1137              raise TypeError("numtypes argument must be a 2-tuple or list "
 
1138                                "of 2-tuples or lists")

1139  
 
1140  
 


1141 -    def __call__(self, arg):


1142          if isinstance(arg, _seq_types): 
1143              try: 
1144                  return self.fn(arg) 
1145              except: 
1146                  return array([self.fn(v) for v in arg]) 
1147          else: 
1148              return self.fn(arg)

1149  
 
1150  
 


1151 -    def __getstate__(self):


1152          d = copy.copy(self.__dict__) 
1153          # remove reference to Cfunc types by converting to strings
 
1154          d['types'] = (_num_type2name[self.types[0]],
 
1155                        _num_type2name[self.types[1]]) 
1156          return d

1157  
 
1158  
 


1159 -    def __setstate__(self, state):


1160          self.__dict__.update(state) 
1161          # reinstate Cfunc types
 
1162          self.types = (_num_name2type[self.types[0]],
 
1163                        _num_name2type[self.types[1]])

1164  
 
1165  
 
1166  # ---------------------------------------------------------------------
 
1167  
 
1168  
 


1169 -def isinputcts(obj):


1170      if isinstance(obj, Variable): 
1171          if obj.defined: 
1172              if compareNumTypes(obj.indepvartype, float64): 
1173                  return isinstance(obj.indepdomain, Interval) and not \
 
1174                         isinstance(obj.output, Pointset) 
1175              elif compareNumTypes(obj.indepvartype, int32): 
1176                  return False 
1177              else: 
1178                  raise TypeError("Unsupported independent variable type for Variable") 
1179          else: 
1180              raise ValueError("Variable is not fully defined") 
1181      else: 
1182          # provide support for e.g. Trajectories. Cannot use Trajectory class
 
1183          # name explicitly here because will run into an infinite import loop
 
1184          # between Variable and Trajectory!
 
1185          if compareNumTypes(obj.indepvartype, float64): 
1186              return isinstance(obj.indepdomain, Interval)

1187  
 
1188  
 


1189 -def isinputdiscrete(var):


1190      return not isinputcts(var)

1191  
 
1192  ##def isinputdiscrete(var):
 
1193  ##    if compareNumTypes(var.indepvartype, float64):
 
1194  ##        return type(var.indepdomain) == ndarray or \
 
1195  ##               isinstance(var.output, Pointset)
 
1196  ##    elif compareNumTypes(var.indepvartype, int32):
 
1197  ##        return True
 
1198  ##    else:
 
1199  ##        raise TypeError("Unsupported independent variable type for Variable")
 
1200  
 
1201  
 


1202 -def isoutputcts(var):


1203      assert isinstance(var, Variable), "Argument must be a Variable" 
1204      if var.defined: 
1205          if compareNumTypes(var.coordtype, float64): 
1206              return isinstance(var.depdomain, Interval) and not \
 
1207                     isinstance(var.output, Pointset) 
1208          elif compareNumTypes(var.coordtype, int32): 
1209              return False 
1210          else: 
1211              raise TypeError("Unsupported dependent variable type for Variable") 
1212      else: 
1213          raise ValueError("Variable is not fully defined")

1214  
 
1215  
 


1216 -def isoutputdiscrete(obj):


1217      return not isoutputcts(obj)

1218  
 
1219  
 


1220 -def iscontinuous(var):


1221      """Determine if variable is continuously defined on its input and
 
1222      output domains.""" 
1223      assert isinstance(var, Variable), "Argument must be a Variable" 
1224      return isinputcts(var) and isoutputcts(var)

1225  
 
1226  
 


1227 -def isdiscrete(var):


1228      """Determine if variable is discretely defined on its input and
 
1229      output domains.""" 
1230      return not (isinputcts(var) and isoutputcts(var))

1231
```

  


| Home | Trees | Indices | Help | PyDSTool |
| --- | --- | --- | --- | --- |

|  |  |
| --- | --- |
| Generated by Epydoc 3.0.1 on Fri May 4 15:24:13 2012 | http://epydoc.sourceforge.net |
